# Enhanced prediction of gene mutation and risk stratification in non-small-cell lung cancer through dual-pathway fusion of radiomics and pathomics

**DOI:** 10.3389/fonc.2025.1646851

**Published:** 2025-09-03

**Authors:** Yang Ji, Ruisheng Ge, Wei Meng, Xinxin Yang, Hongguang Lu, Bei Lu, Chenglong Zheng, Yang Teng, Hong Pan, Ling Liu, Jiaheng Xu, Tong Zhang

**Affiliations:** ^1^ Department of Radiology, Fourth Affiliated Hospital of Harbin Medical University, Harbin, China; ^2^ Department of Pathology, Fourth Affiliated Hospital of Harbin Medical University, Harbin, China; ^3^ Department of Radiology, Harbin Medical University Cancer Hospital, Harbin, China; ^4^ Precision Medical Center, Department of Pathology, Harbin Medical University Cancer Hospital, Harbin, China; ^5^ Department of Thoracic Surgery, Fourth Affiliated Hospital of Harbin Medical University, Harbin, China; ^6^ Department of Medical Oncology, Fourth Affiliated Hospital of Harbin Medical University, Harbin, China

**Keywords:** epidermal growth factor receptor (EGFR), non-small-cell lung cancer (NSCLC), radiomics, pathomics, deep learning, risk stratification

## Abstract

**Purpose:**

This study aimed to develop and validate a multimodal combined model that integrated radiomics, pathomics and clinical features to precisely predict EGFR status and risk stratification in NSCLC.

**Materials and methods:**

We retrospectively analyzed 387 patients with NSCLC from two hospitals (the train cohort: n=193; the internal validation cohort: n=83; the external validation cohort: n=111). Radiomics models were developed using 3D CNN for the construction of deep learning radiomics (DLRadiomics). Weakly supervised learning and multi-instance learning were used to develop pathomics signature (Pathomics). We conducted an in-depth analysis of clinical features resulting in a clinical signature (Clinical). Finally, we integrated them into a comprehensive nomogram-Nomogram. The comparative analysis of all models was conducted through a comprehensive evaluation. The distribution of predictive features for Nomogram across different EGFR mutation subtypes was evaluated. The Kaplan-Meier curve was employed to assess the predictive capability of Nomogram in risk stratification among cases with survival outcomes.

**Results:**

In comparison to Clinical, DLRadiomics and Pathomics models, Nomogram exhibits superior predictive performance (the train cohort: AUC=0.986, 95%CI=0.969-1.000; the internal validation cohort: AUC=0.796, 95%CI=0.659-0.932; the external test cohort: AUC=0.850, 95%CI=0.719-0.981). Nomogram could also be used to predict effectively EGFR mutation subtype (P<0.05). In the validation and test cohorts, the log rank test proved the effectiveness of Nomogram in predicting risk stratification (P<0.05).

**Conclusions:**

We demonstrated that Nomogram which integrated radiomics, pathomics and clinical features, could be served as a noninvasive and reusable tool to precisely predict EGFR status and risk stratification in NSCLC.

## Introduction

The latest cancer statistics indicate that lung cancer remains the primary cause of cancer-related deaths, particularly among individuals aged 50 and above. The annual number of fatalities due to lung cancer significantly outnumbers the combined totals of those from colorectal, breast, and prostate cancers (1, 2). The proportion of non-small cell lung cancer (NSCLC) in lung cancer is approximately 80% (3). The rapid advancement of personalized precision therapy has increasingly benefited patients with NSCLC, markedly enhancing their prognosis (4, 5). Epidermal growth factor receptor (EGFR) is expressed in approximately 50% of NSCLCs, and its expression correlates with poor prognosis (6). Multiple studies have demonstrated that the administration of EGFR-tyrosine kinase inhibitors (TKIs) significantly extends the survival duration of EGFR-mutated patients (7). In addition, tumors with EGFR mutations usually have a low response to immune checkpoint inhibitors used in immunotherapy (8). Therefore, identifying the EGFR mutation status of NSCLC patients prior to treatment is crucial for formulating clinical treatment decisions. For a long time, the identification of EGFR mutation status primarily relied on genetic testing of tissue biopsies or surgical specimens. However, tumor tissue exhibits heterogeneity, meaning that biopsy tissue cannot comprehensively represent the entire tumor. Biopsies have inherent limitations, including inadequate quantity and quality of tissue sampling, as well as posing a potential risk of tumor metastasis (9). EGFR mutation status can alter with disease progression or therapeutic intervention, such as the emergence of drug resistance being associated with mutations in EGFR exon 20. Repeated biopsies for advanced patients are impractical and challenging for their tolerance. Furthermore, the substantial costs associated with genetic testing increase the financial burden on patients. There is an urgent demand for a precise, swift, reusable, and cost-effective prediction tool capable of forecasting EGFR mutation status in NSCLC prior to treatment, thereby guiding personalized therapeutic decisions.

Early researchers utilized clinical data and computerized tomography (CT) images morphological signs to predict EGFR mutation status, including the absence of smoking, pleural indentation, and the appearance of ground glass (GGO) in lesions, all of which were believed to be closely linked to EGFR mutations (10–12). Radiomics enables the prediction of a range of clinical outcomes, including tumor pathological classification, recurrence, progression, metastasis, and survival prognosis, by leveraging high-throughput extraction of medical image features and subsequently employing machine learning algorithms to construct models (13–15). The advantage of radiomics lies in its ability to noninvasively and conveniently extract quantitative features associated with tumor biological phenotypes, thereby comprehensively assessing tumor heterogeneity. With the advancement of artificial intelligence technology, deep learning demonstrates a more effective capability compared to traditional radiomics (16, 17). In the prediction of EGFR mutation status, many researchers were focused on 2D deep learning based on the largest cross-sectional level of the lesion, while infrequently used 3D convolutional neural networks (CNNs), potentially compromising the capture of comprehensive and three-dimensional spatial position information of the lesion (18, 19).

In recent years, the integration of artificial intelligence with digital pathology has yielded encouraging results. Pathomics enables the extraction of tumor region features from pathological images at the microscopic level, capturing information on diverse tissue phenotypes that are invisible to the naked eye. These intricate visual features, extracted from high-resolution histopathological images, have demonstrated promising prospects in predicting various aspects of malignant lesions, including their staging, pathological subtypes, lymph node metastasis status, and treatment response (20–22). However, most studies tend to adopt supervised classification learning methods that require manual annotation, resulting in significant time and labor expenditure. Theoretically, the presence of EGFR mutations in NSCLC could influence the microscopic characteristics and growth patterns of tumor cells, and these features can be captured through pathomics approaches. The integration of radiomics and pathomics enables the extraction of multi-dimensional features from both macro and micro perspectives, providing a comprehensive view of the biological information of tumors and enhancing the predictive capabilities of existing models. To the best of our knowledge, there is no relevant study available through the fusion of radiomics and pathomics to predict EGFR mutation status in NSCLC. Our objective is to develop and validate pathomics models leveraging weakly supervised deep learning and multi-instance learning (MIL) techniques without manual annotation. By integrating this model with 3D CNN-based radiomics and clinical features to build a multimodal combination model to predict the EGFR mutation status and risk stratification in NSCLC. It serves as a precise, rapid, economical, and reusable prediction tool, aiding in clinical decision-making for personalized precision treatment.

## Materials and methods

### Patient cohort and data collection

This study was approved by the institutional review board of the Fourth Affiliated Hospital of Harbin Medical University (NO.2024-LLSC-21). Informed consent to participate was waived by the IRB because it was a retrospective study and the data used for the study had hidden personal information. Patients with histologically confirmed NSCLC who underwent surgery or biopsy in the Fourth Affiliated Hospital of Harbin Medical University from June 2020 to November 2023 were retrospectively collected. These patients were considered as the primary cohort for this study. The inclusion criteria were as follows: (a) Thin slice CT images within 14 days before surgical resection or biopsy were available in picture archiving and communication systems (PACS). (b) Formalin fixed paraffin specimens (FFPE) of surgically resected or biopsied tumor tissues were subjected to next-generation sequencing (NGS) to obtain EGFR mutation status. (c) Pathological tissue slides with hematoxylin and eosin (H&E) staining were available for whole slide image (WSI) digital scanning. (d) The corresponding clinical data could be queried in the electronic medical record system. The exclusion criteria were: (a) Lack of preoperative or pre-treatment CT images in PACS. (b) Poor CT image quality or presence of artifacts. (c) Difficult to segment region of interest (ROI) due to factors such as atelectasis. (d) Lack of pathological tissue sections or poor staining quality. (e) Lost or incomplete clinical data.

**Figure 1:**
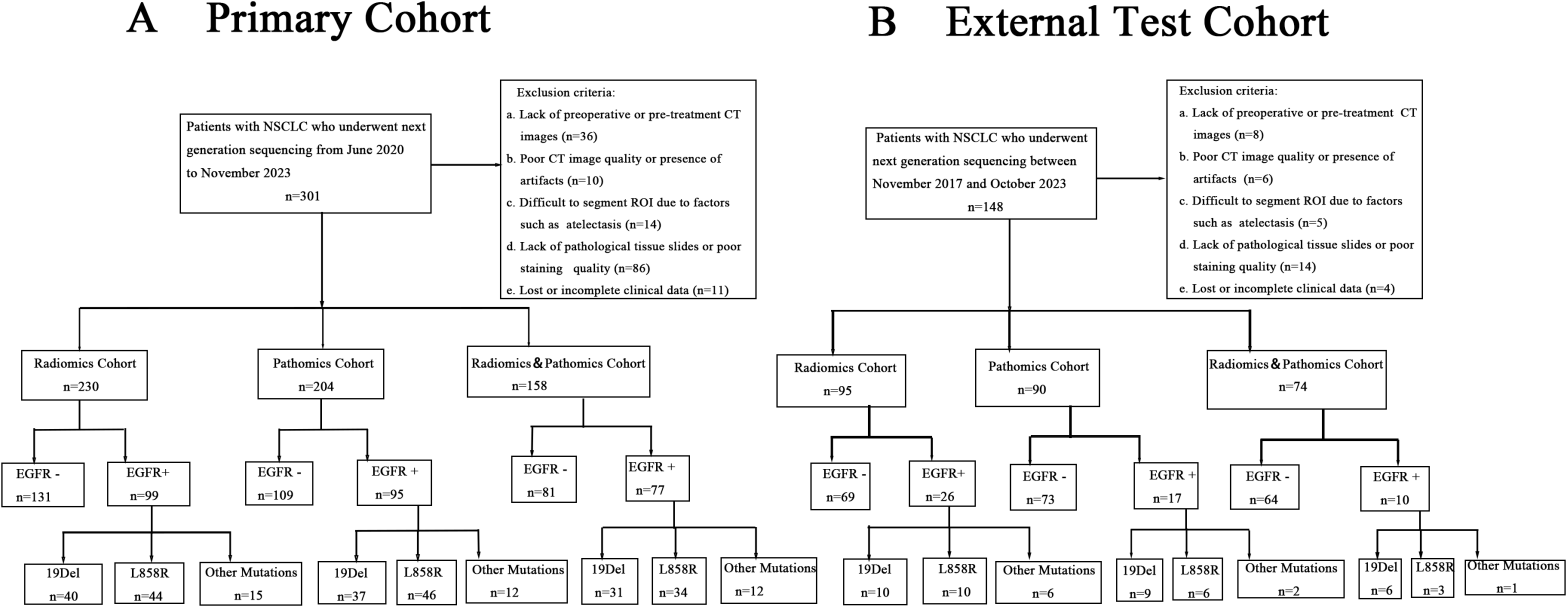
Patient flow diagram of our study. **(A)** Primary cohort. **(B)** External test cohort.

In the primary cohort, we divided the entire dataset of 276 cases into a training and internal validation set with a 7:3 split, resulting in 193 cases for training and 83 cases for internal validating. Of these, patients with CT images were categorized into the Radiomics Cohort (n=230) for the purpose of developing radiomics models. Cases with pathological tissue slice images were selected as Pathomics Cohort (n=204), which was used to develop pathomics models. 158 patients (115 in the training set and 43 in the internal validation set) had both CT images and pathologic data available, which were included in the Radiomics&Pathomics Cohort (n=158). As we all know, in the real-world clinical setting, CT scans and tissue biopsies are not always performed at the same medical facility. This division was conducted to thoroughly validate the effectiveness of the multi-omics fusion algorithm. From November 2017 to October 2023, a total of 111 NSCLC patients from Harbin Medical University Cancer Hospital were enrolled in the external test cohort, comprising the Radiomics Cohort (n=95), the Pathomics Cohort (n=90), and the Radiomics&Pathomics Cohort (n=74). The criteria for patient selection remained consistent with those applied to the primary cohort. The specific details of the patient flow diagram can be found in **Figure 1**.

### Image acquisition and segmentation

CT images used in the primary cohort were from five suppliers (GE, Canon, Siemens, Philips, and United Imaging). Meanwhile, the external test cohort comprises equipment from three of these manufacturers (GE, Siemens, and Philips), thereby guaranteeing the versatility of the developed model across various scanning devices. The resolution of all CT scans was 512×512, with the axial slice spacing ranging from 0.625 to 1.5mm. All thin-layer CT images were imported into ITK-SNAP software (version 3.8.0, www.itksnap.org) in DICOM format for annotation. A radiologist with eight years’ working experience manually delineated the region of interest of tumor lesions layer by layer in the lung window setting. Another radiologist was responsible for the examination and correction, and a radiologist with 20 years’ experience was consulted to reach a consensus when there was a disagreement.

The H&E stained pathological slides of all patients with NSCLC in the primary cohort were scanned by the digital scanner (kf-pro-020) at 40× magnification (0.125μm/pixel) provided by KONFOONG BIOINFORMATION TECH CO., LTD (http://www.kfbiopathology.com) to obtain the WSIs. The pixel size was 7.5μm. The original format of WSI was KFB, which was converted to SVS format by converter for subsequent pathomics models development. The digital scanner used by the external test cohort was Leica APERIO CS2, which operated at a magnification of 20×.

**Figure 2:**
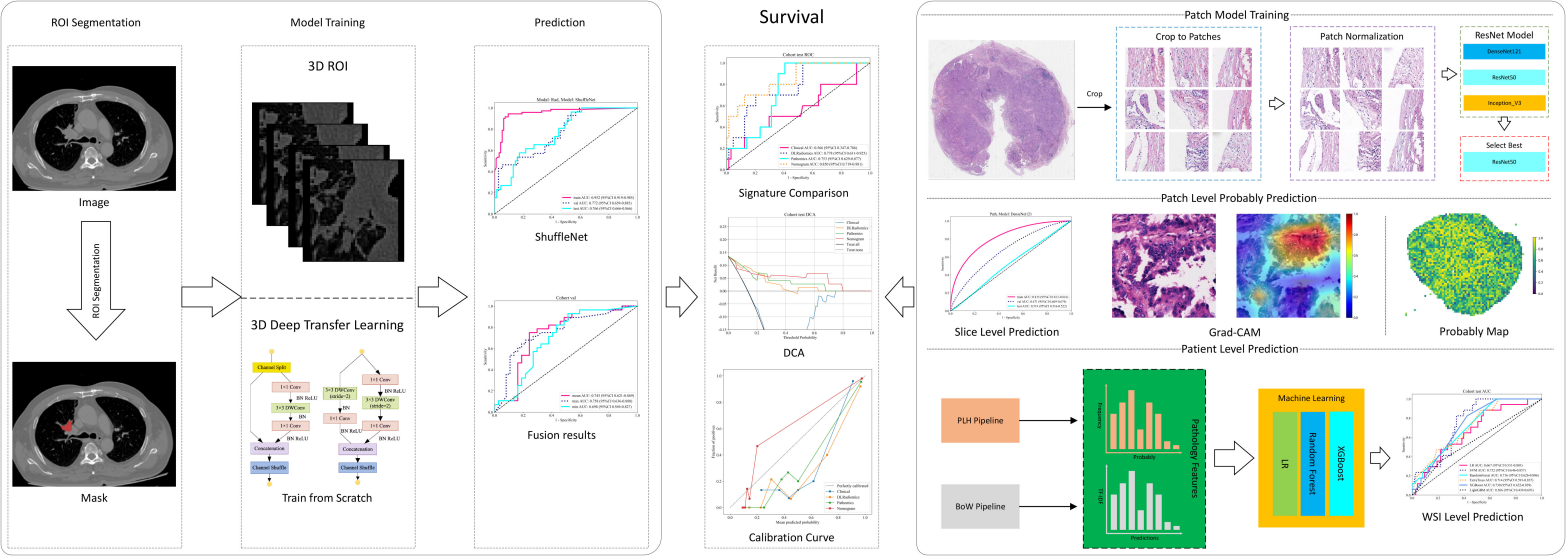
Workflow of our study. ROI, region of interest; WSI, whole slide image; DCA, decision curve analysis; LR, logistic regression; DLRadiomics, deep learning radiomics; BoW, bag of words.

The EGFR mutation status was determined to be wild-type or mutant-type through NGS. The mutation subtypes included exon 19 deletions (19del), the point mutation of exon 21 (L858R) and other mutations. These genetic test results were regarded as the gold standard of this study. The clinical features collected included age, sex, smoking history, family history of cancer, histological type, stage, lesion location, and lesion maximum diameter. The imaging morphological features, such as lesion type, lobulation sign, spiculation sign, pleural indentation sign, vacuole or cavity sign, were also involved in this study. **Figure 2** delineates the whole analysis workflow.

### Deep learning radiomics

We employed 3D deep learning to identify EGFR mutation status in the ROI of the patients with NSCLC. To ensure consistency in voxel spacing across different volumes of interest (VOI), we used a fixed resolution resampling method for spatial normalization. This process standardized the voxel spacing to 1*mm* ×1*mm* ×1*mm*, allowing for accurate comparisons and evaluations by aligning spatial dimensions across images. Unlike with 2D CNNs, we cropped the entire ROI area to obtain the smallest bounding cuboid, which was then used for training. To enhance the robustness of our model, we applied Z-score normalization to the images, which were then utilized as inputs. For the cropped regions, we employed ResNet50-3D, ShuffleNet-3D, and Densenet121-3D models to predict their EGFR mutation status. For the input data of these models, we performed a min-max transformation to normalize the gray values, scaling them to a range between -1 and 1. Additionally, we resized each cropped subregion image to 96×96×96 using nearest interpolation, which served as the input for our 3D CNN models. During the training process, we adopted real-time data augmentation techniques, including random cropping, random 90° rotations, and random flipping along the x, y, and z axes, to increase the diversity of the training data. For test images, however, only normalization was performed to maintain consistency in data preprocessing. The predicted probabilities from the CNN models were designated as the Deep Learning Radiomics Signature. Our study included three different models, for which we trained a separate model for each. To understand the distinct characteristics of each modality, we conducted a detailed comparative analysis of the results from each. Additionally, to explore the integration of multimodal approaches, we combined their results using three different methods: maximum, minimum, and mean value fusion. The final prediction results from the model were then used as the deep learning radiomics signature (DLRadiomics). The Radiomics training details were in **Supplementary 1A**.

### Pathomics

First, given the large size of WSIs, which typically measure around 100,000×50,000 pixels, we acquired these images at a 20× magnification, resulting in a pixel resolution of approximately 0.5μm/pixel. We then divided the WSIs into smaller patches of 512×512 pixels and used the OnekeyAI OKT-patch2 predict tool to remove all white backgrounds, yielding over 40,000 distinct, non-overlapping tiles. We developed a pathomics approach to identify EGFR mutation status in NSCLC by analyzing WSIs at the patch level. The process involved extracting relevant features through a weakly supervised learning approach and aggregating these features to construct a predictive model for EGFR mutation status.

#### Weakly supervised learning

Our approach utilized deep learning to generate predictions at both the patch and WSI levels. We segmented WSIs into smaller patches, with all patches from a single sample sharing the same EGFR label. For patch-level prediction, we evaluated three top-performing networks: DenseNet121, ResNet50, and Inception_v3. The goal was to assess the likelihood of each patch being classified into a category corresponding to its WSI. In our pathology model training, we implemented online data augmentation and normalization strategies similar to those used in the imaging model. These strategies included random horizontal and vertical flips of the patches to increase the diversity of the training data, center cropping to 224 × 224 pixels (and 299 × 299 pixels for InceptionV3) to ensure uniform input size, and Z-score normalization across RGB channels to standardize the distribution of pixel values and ensure consistency and robustness in the model’s performance. Further details on the training process can be found in **Supplementary Material 2A**.

#### Multi-instance learning for WSI integration

Following the training of deep learning model, we focused on predicting labels and their associated probabilities for individual patches. These probabilities were then aggregated using a classifier to derive WSI-level predictions. We employed a multi-instance learning-based approach for feature fusion, aiming to enhance the predictive accuracy of our models. This method involves integrating various data points or instances from a single sample to formulate a comprehensive feature set. To effectively integrate patch probabilities, two unique machine learning methodologies namely histogram feature aggregation and bag of words feature aggregation were developed, detailed in **Supplementary Material 2B**. These methodologies allowed us to construct a comprehensive model for predicting EGFR mutations in lung cancer based on pathomics analysis.

#### Pathomics signature

We developed a comprehensive pathomics signature by amalgamating patch-level predictions, probability histograms, and term frequency-inverse document frequency (TF-IDF) features to construct individualized patient profiles. To refine feature selection, we utilized the Pearson correlation coefficient, retaining only one feature from each pair with a correlation exceeding 0.9. The model incorporated various machine learning algorithms, including logistic regression (LR), support vector machine (SVM), random forest (RF), and extreme gradient boosting (XGBoost), and was referred to as the Pathomics Signature.

### Multiomics fusion and model evaluation

We employed univariate and multivariate regression analyses to examine clinical features, retaining those with a p-value < 0.05 for inclusion in the combined model. We utilized models such as LR, SVM, and RF to construct the clinical model. The predictive probabilities of this model were defined as the Clinical Signature. In the final stage, we integrated the clinical signature, DLRadiomics signature, and pathomics signature into a comprehensive nomogram, referred to as the combined model (Nomogram). In the Radiomics & Pathomics cohorts, a comparative analysis was conducted to assess the predictive performance of the multi-omics fusion model against the single-mode model. We evaluated the predictive performance of the Nomogram, Clinical, DLRadiomics, and Pathomics models for EGFR mutation status using AUC, accuracy, sensitivity, specificity, PPV, and NPV collectively. Calibration curves and decision curve analysis (DCA) were used to validate the clinical benefits of each model. The optimal cut-off value for survival samples was determined using Nomogram based on xtile. Subsequently, we categorized them into high-risk and low-risk groups, and employed the Kaplan-Meier curve to assess the model.

### Statistical analysis

All statistical analyses were performed using OneKeyAI v2.2.3 software. To compare the clinical characteristics of patients, we conducted a series of statistical tests, including the anova for continuous variables and the Chi-square test for discrete variables. A p-value of less than 0.05 was considered to indicate a statistically significant difference.

For the analysis, we employed a range of software tools and custom code to ensure precision and efficiency. Computational models and data analysis were primarily implemented in Python v3.7.12. Key Python libraries used include PyTorch v1.8.0 for deep learning algorithms, OnekeyAI v2.2.3 for patch extraction and processing, scikit-learn v1.0.2 for machine learning models, and PyRadiomics v3.0 for extracting radiomic features. All the codes used in this study are available at the following link (https://github.com/OnekeyAI-Platform/MMF).

### Hardware configuration

The deep learning models in this study were trained using robust hardware: an Intel 14900k CPU, 64GB RAM, and an NVIDIA RTX 4090 GPU. The system operated on Windows 11. Analytical work was conducted using Python version 3.7.12 and stats models version 0.13.2, and machine learning model development utilized the scikit-learn version 1.0.2 interface.

## Results

### Baseline characteristics

Our retrospective study encompassed a total of 387 patients with NSCLC, comprising 193 patients in the training set, 83 in the internal validation set, and 111 in the external test set. **Table 1** presents the baseline characteristics of all cohorts. The results indicated no significant difference in clinical characteristics among the three groups (p value>0.05), thereby validating the effectiveness of our random allocation.

**Table 1:** Baseline characteristics of our cohort.

Characteristics	Train cohort (n=193)	Validation cohort (n=83)	Test cohort (n=111)	P value
Age (years)	63.104 ± 8.873	64.108 ± 8.626	57.973 ± 9.966	0.385
Sex				0.597
Female	106 (54.922)	42 (50.602)	44 (39.640)	
Male	87 (45.078)	41 (49.398)	67 (60.360)	
Smoking History				0.482
Never	131 (67.876)	52 (62.651)	61 (54.955)	
Current or former	62 (32.124)	31 (37.349)	50 (45.045)	
Family history of cancer				0.424
No	174 (90.155)	78 (93.976)	84 (75.676)	
Yes	19 (9.845)	5 (6.024)	27 (24.324)	
Histological type				0.463
Adenocarcinoma	171 (88.601)	69 (83.133)	97 (87.387)	
Squamous cell carcinoma	17 (8.808)	11 (13.253)	9 (8.108)	
Other NSCLC	5 (2.591)	3 (3.614)	5 (4.505)	
Stage				0.619
I	81 (41.969)	42 (50.602)	12 (10.811)	
II	26 (13.472)	9 (10.843)	11 (9.910)	
III	57 (29.534)	21 (25.301)	25 (22.523)	
IV	29 (15.026)	11 (13.253)	63 (56.757)	
Lesion location				0.428
RUL	67 (34.715)	27 (32.530)	40 (36.036)	
RML	12 (6.218)	6 (7.229)	2 (1.802)	
RLL	38 (19.689)	15 (18.072)	16 (14.414)	
LUL	39 (20.207)	12 (14.458)	22 (19.820)	
LLL	28 (14.508)	13 (15.663)	21 (18.919)	
Hilum	7 (3.627)	8 (9.639)	0 (0.000)	
Mixed	2 (1.036)	2 (2.410)	10 (9.009)	
Lesion type				0.346
Solid	136 (70.466)	55 (66.265)	111 (100.000)	
Part-solid	42 (21.762)	24 (28.916)	0 (0.000)	
Ground-glass	15 (7.772)	4 (4.819)	0 (0.000)	
Lesion maximum diameter (cm)	3.128 ± 1.888	3.134 ± 1.950	3.806 ± 1.712	0.787
Lobulation sign				1.0
No	27 (13.990)	12 (14.458)	17 (15.315)	
Yes	166 (86.010)	71 (85.542)	94 (84.685)	
Spiculation sign				0.346
No	57 (29.534)	30 (36.145)	12 (10.811)	
Yes	136 (70.466)	53 (63.855)	99 (89.189)	
Pleural indentation sign				0.196
No	30 (15.544)	19 (22.892)	26 (23.423)	
Yes	163 (84.456)	64 (77.108)	85 (76.577)	
Vacuole or Cavity sign				0.238
No	114 (59.067)	56 (67.470)	73 (65.766)	
Yes	79 (40.933)	27 (32.530)	38 (34.234)	

RUL, right upper lobe; RML, right middle lobe; RLL, right lower lobe; LUL, left upper lobe; LLL, left lower lobe.

### Clinical signature

**Figure 3:**
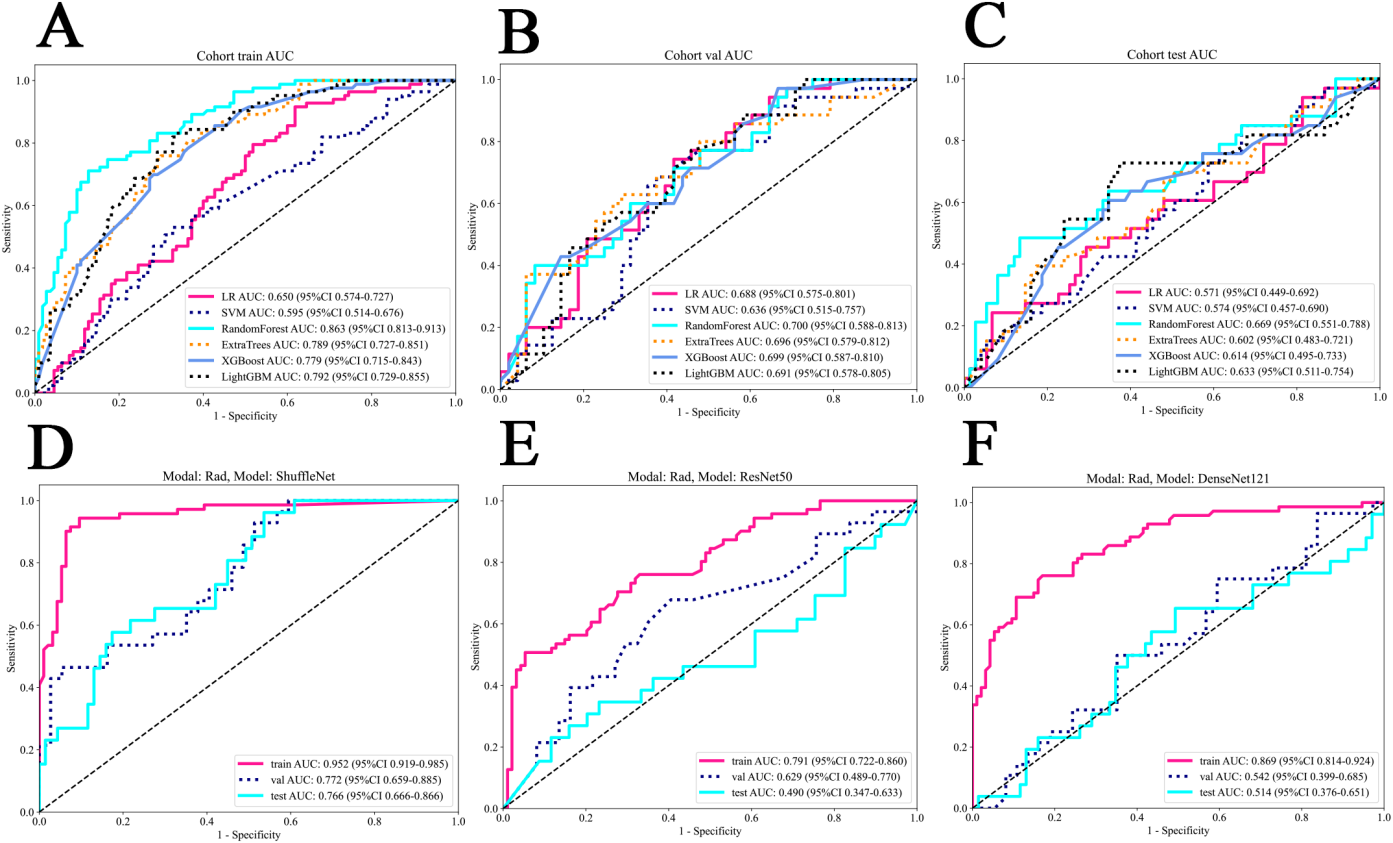
The ROC of all cohorts for clinical and deep learning radiomics models. **(A)** The train cohort of clinical models. **(B)** The internal validation cohort of clinical models. **(C)** The external test cohort of clinical models. **(D)** ShuffleNet model for deep learning radiomics. **(E)** ResNet50 model for deep learning radiomics. **(F)** DenseNet121 model for deep learning radiomics. LR, logistic regression; SVM, support vector machine.

We assessed the clinical features using both univariable and multivariable models, calculating the odds ratio (OR) and corresponding p-value for each feature. Thirteen clinical features were incorporated into the construction of the combined model. Based on univariate analysis, Sex and Smoking history were found to be significantly associated with EGFR mutation status (P<0.05). These features were included in the subsequent multivariable analysis. After multivariable analysis, only Smoking history variable (OR:0.819; 95%CI:0.714-0.940; P_value:0.017) exhibited a significant negative correlation with EGFR mutation status, indicating a higher probability of EGFR mutation positivity among non-smoking patients (**Table 2**). The RF model demonstrated superior performance, achieving the highest area under the receiver operating characteristic curve (AUC) of 0.863;95% confidence intervals (CI) was 0.813 - 0.913 in the training set, 0.700 (95% CI: 0.588-0.813) in the internal validation set and 0.669 (95% CI: 0.551 - 0.788) in the external test set. **Figures 3A–C** present the receiver operating characteristic curve (ROC) of all cohorts for multiple models of clinical features.

**Table 2:** The OR and P value of clinical features in univariable and multivariable model.

Clinical features	OR	OR lower 95%CI	OR upper 95%CI	p_value	OR	OR lower 95%CI	OR upper 95%CI	P value
Age	0.998	0.992	1.005	0.664				
Sex	0.821	0.731	0.923	0.006	0.893	0.785	1.015	0.147
Smoking History	0.776	0.686	0.878	0.001	0.819	0.714	0.940	0.017
Family history of cancer	0.881	0.723	1.074	0.292				
Histological type	0.822	0.689	0.981	0.069				
Stage	0.970	0.920	1.021	0.331				
Lesion location	0.999	0.969	1.028	0.931				
Lesion type	1.050	0.938	1.176	0.472				
Lesion maximum diameter	0.976	0.946	1.007	0.207				
Lobulation sign	0.983	0.829	1.166	0.871				
Spiculation sign	0.964	0.846	1.097	0.638				
Pleural indentation sign	1.036	0.880	1.220	0.719				
Vacuole or Cavity sign	1.001	0.887	1.129	0.994				

OR, odds Ratio; CI, confidence intervals.

### Deep learning radiomics signature

The ShuffleNet model demonstrated superior performance, achieving the highest AUC of 0.952 (95% CI: 0.919-0.985) in the training cohort, 0.772 (95% CI: 0.659-0.885) in the internal validation cohort and 0.766 (95% CI:0.666-0.866) in the external test cohort. The multi-model fusion results did not outperforming the single model performance (detailed in **Supplementary Table S1** and **Supplementary Figure S1** in **Supplementary 1B**), we opted to use the ShuffleNet results for the final comparative analysis. **Figures 3D–F** show the performance of three models.

### Pathomics signature

**Figure 4:**
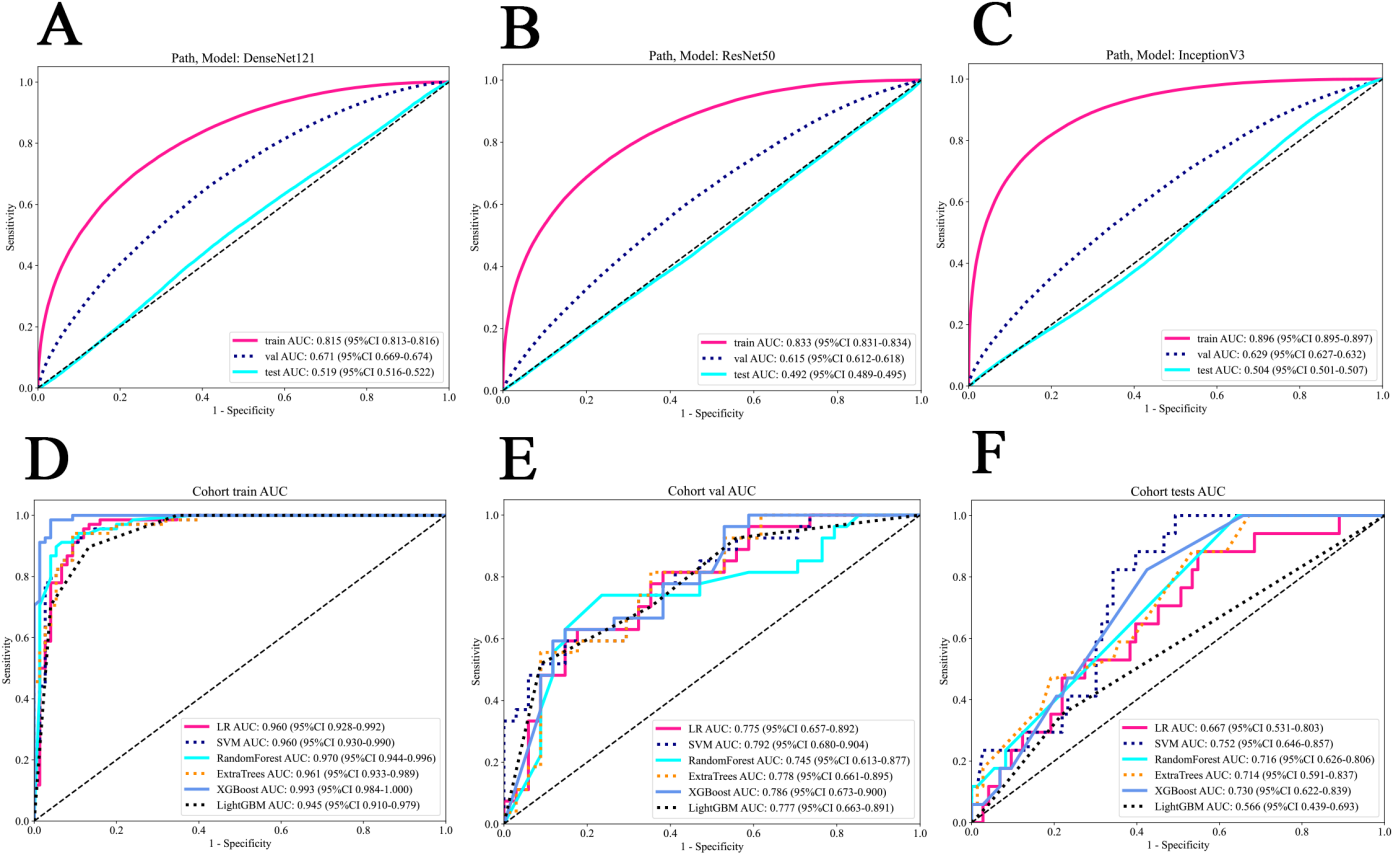
The ROC of various models in patch and WSI level prediction. **(A)** DenseNet121 model. **(B)** Resnet50 model. **(C)** Inception V3 model. **(D)** all models in the train cohort. **(E)** all models in the internal validation cohort. **(F)** all models in the external test cohort. WSI, whole slide image.

**Figure 5:**
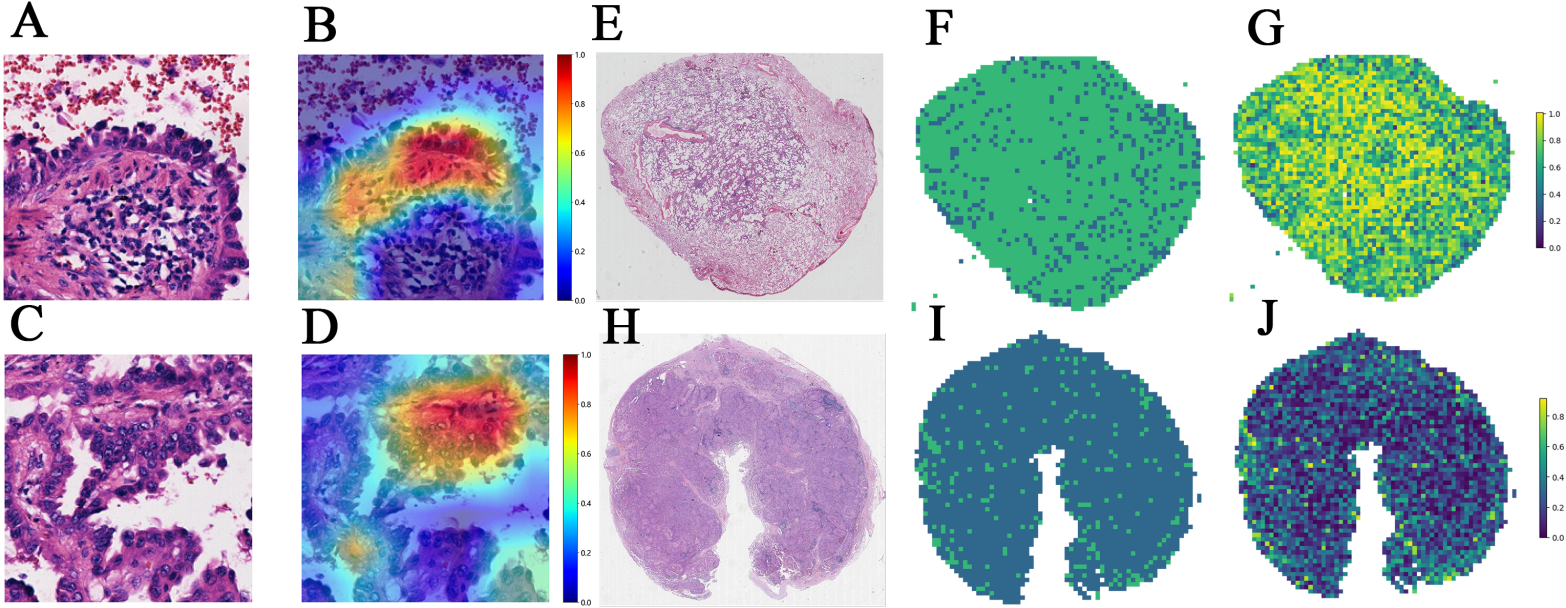
**(A–D)** The Grad-CAMs of patches in predicting EGFR status in a 49-year-old female with RUL (right upper lobe) invasive adenocarcinoma and Stage II B. **(A)** Patch 1 image. **(B)** Patch 1's corresponding Grad-CAM. **(C)** Patch 2 image of the same patient. **(D)** Patch 2's corresponding Grad-CAM. **(E–J)** Visualization of WSI-level predictions. **(E)** WSI in a 47-year-old female with RUL (right upper lobe) invasive adenocarcinoma and Stage I A. **(F)** predicted labels corresponding to **(E)**. **(G)** prediction probability heatmap corresponding to **(E)**. **(H)** WSI in a 71-year-old female with left lung adenocarcinoma and Stage III B. **(I)** predicted labels corresponding to **(H)**. **(J)** prediction probability heatmap corresponding to **(H)**. Grad-CAM: Grad-CAM class localization maps are produced by visualizing the gradients flowing into the final convolutional layer of the network, just before the fully connected layers. This layer is optimal for map generation as it retains class-specific spatial information from the input image, which is lost in the fully connected layers. A key advantage of Grad-CAM over conventional CAMs is that it does not require any modifications to the existing model architecture or retraining of the model. The last convolutional layer of the network was made transparent to the prediction of the response, as illustrated.

In patch level prediction, The DenseNet121 model was highly efficient (internal validation cohort: AUC=0.671; 95% CI=0.669-0.674), highlighting its value in precise pathological analysis (**Figures 4A–C**). The gradient-weighted class activation mappings (Grad-CAMs) of patches can be found in **Figures 5A–D**.

In WSI level prediction, using a manner akin to radiomics, we extracted 206 features through multi-instance learning, with bag of words (Bow) and probability of label histogram (PLH) features contributing 103 features each. Additionally, probability features and label features contributed 2 and 101 features, respectively. We utilized correlation coefficients and Lasso regression for feature selection, ultimately identifying 10 pathomics features.

The SVM model exhibited outstanding performance, achieving the highest AUC of 0.960 (95% CI: 0.930 – 0.990) in the training cohort, 0.792 (95% CI: 0.680-0.904) in the internal validation cohort and 0.752 (95% CI: 0.646 - 0.857) in the testing cohort (**Figures 4D–F**). **Figures 5E–J** show visualization of prediction results for two patients.

### Multiomics fusion

**Figure 6:**
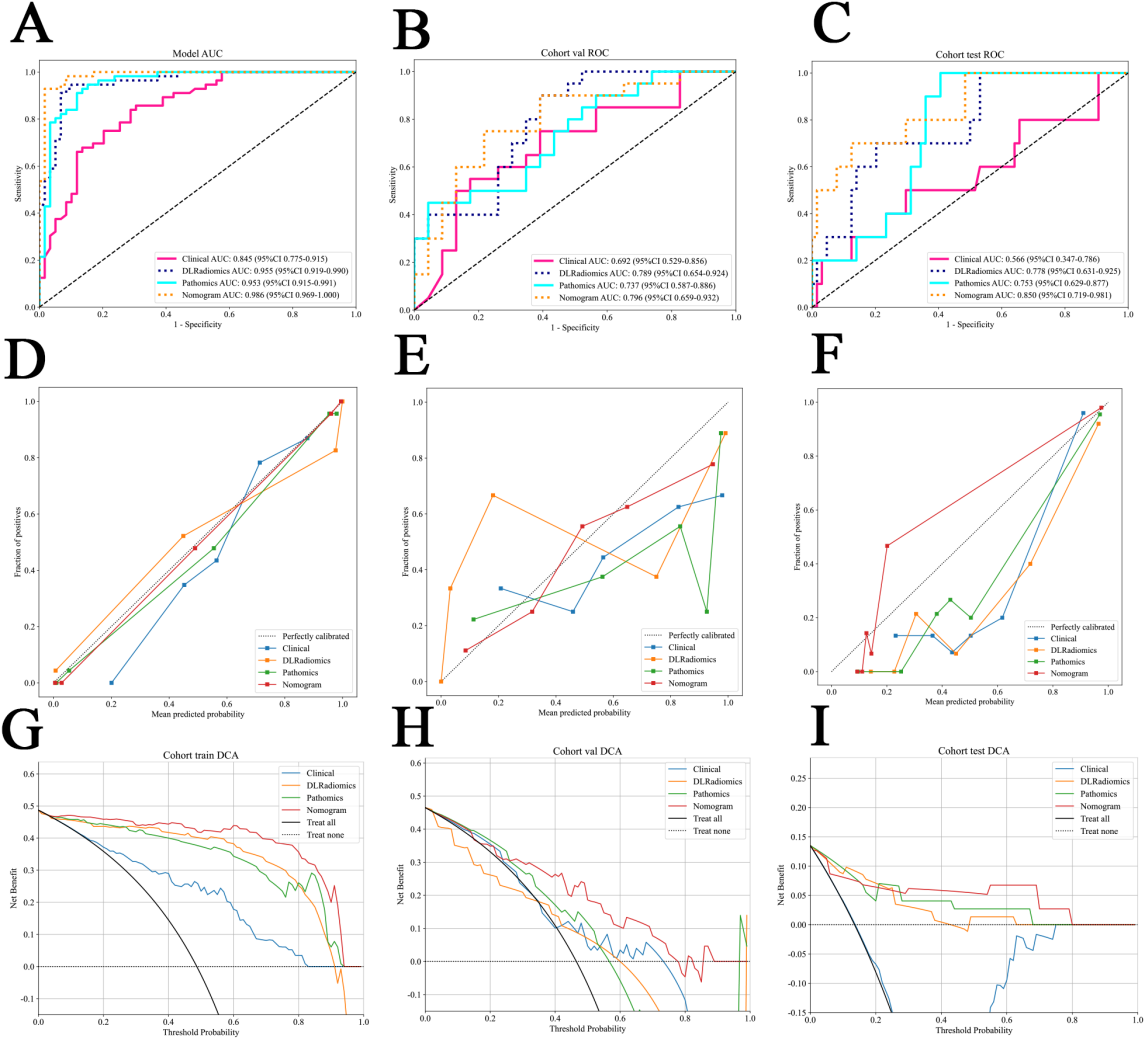
**(A–C)** The ROC for different signatures across various datasets. **(A)** The train cohort. **(B)** The internal validation cohort. **(C)** The external test cohort. **(D–F)** Different signatures’ calibration curve on all cohorts. **(D)** The train cohort. **(E)** The internal validation cohort. **(F)** The external test cohort. G-I The decision curve analysis (DCA) of different signatures for all cohorts. **(G)** The train cohort. **(H)** The internal validation cohort. **(I)** The external test cohort.

In the comparative analysis of model performance, Nomogram demonstrated superior performance, which achieved the highest AUC of 0.796 in the internal validation cohort and 0.850 in the external test cohort. It was evident that through multimodal fusion, the prediction performance had been significantly enhanced. **Table 3** presents a comparative analysis of various models. **Figures 6A–C** show the ROC for different signatures across various datasets.

**Table 3:** The comparison of the capabilities of the models.

Signature	Accuracy	AUC	95% CI	Sensitivity	Specificity	PPV	NPV	Cohort
Clinical	0.765	0.845	0.7751 - 0.9153	0.839	0.695	0.723	0.820	Train
DLRadiomics	0.913	0.955	0.9193 - 0.9902	0.929	0.898	0.897	0.930	Train
Pathomics	0.887	0.953	0.9149 - 0.9906	0.929	0.847	0.852	0.926	Train
Nomogram	0.948	0.986	0.9686 - 1.0000	0.911	0.983	0.981	0.921	Train
Clinical	0.674	0.692	0.5287 - 0.8561	0.500	0.826	0.714	0.655	Val
DLRadiomics	0.721	0.789	0.6540 - 0.9243	0.850	0.609	0.654	0.824	Val
Pathomics	0.698	0.737	0.5874 - 0.8865	0.400	0.957	0.889	0.647	Val
Nomogram	0.744	0.796	0.6589 - 0.9324	0.700	0.783	0.737	0.750	Val
Clinical	0.662	0.566	0.3470 - 0.7858	0.400	0.703	0.174	0.882	Test
DLRadiomics	0.770	0.778	0.6309 - 0.9253	0.600	0.797	0.316	0.927	Test
Pathomics	0.635	0.753	0.6293 - 0.8769	0.900	0.594	0.257	0.974	Test
Nomogram	0.838	0.850	0.7195 - 0.9805	0.600	0.875	0.429	0.933	Test

AUC, area under the receiver operating characteristic curve; CI, confidence intervals; PPV, positive predictive value; NPV, negative predictive value.

We examined the distribution of predictive features for our combined model—Nomogram—across different EGFR mutation subtypes. Through ANOVA statistical analysis, we found that Nomogram has a p-value less than 0.05 in different groups, demonstrating that Nomogram can effectively predict various EGFR mutation status (**Supplementary Table S2** and **Supplementary Figure S2** in **Supplementary Material 3A**).

The calibration curve, evaluated using the Hosmer-Lemeshow (HL) test, assesses the concordance between predicted probabilities and actual outcomes. In our study, Nomogram exhibited impressive calibration, as evidenced by HL test statistics of 0.470, 0.862 and 0.075 for the training, internal validation and external test datasets, respectively. Notably, these values all exceeded the threshold of 0.05, indicating a statistically significant good fit (**Figures 6D–F**). From the DCA results, compared to other signatures, Nomogram exhibits a larger potential for obtaining net benefit (**Figures 6G–I**). For practical clinical application, we integrated clinical features with a p-value < 0.05 in multivariate regression analysis, along with the DLRadiomics Signature and Pathomics Signature, to form Nomogram. This model was visualized through a nomogram for easier interpretation (**Supplementary Figure S3** in **Supplementary Material 3B**).

### Risk stratification

**Figure 7:**
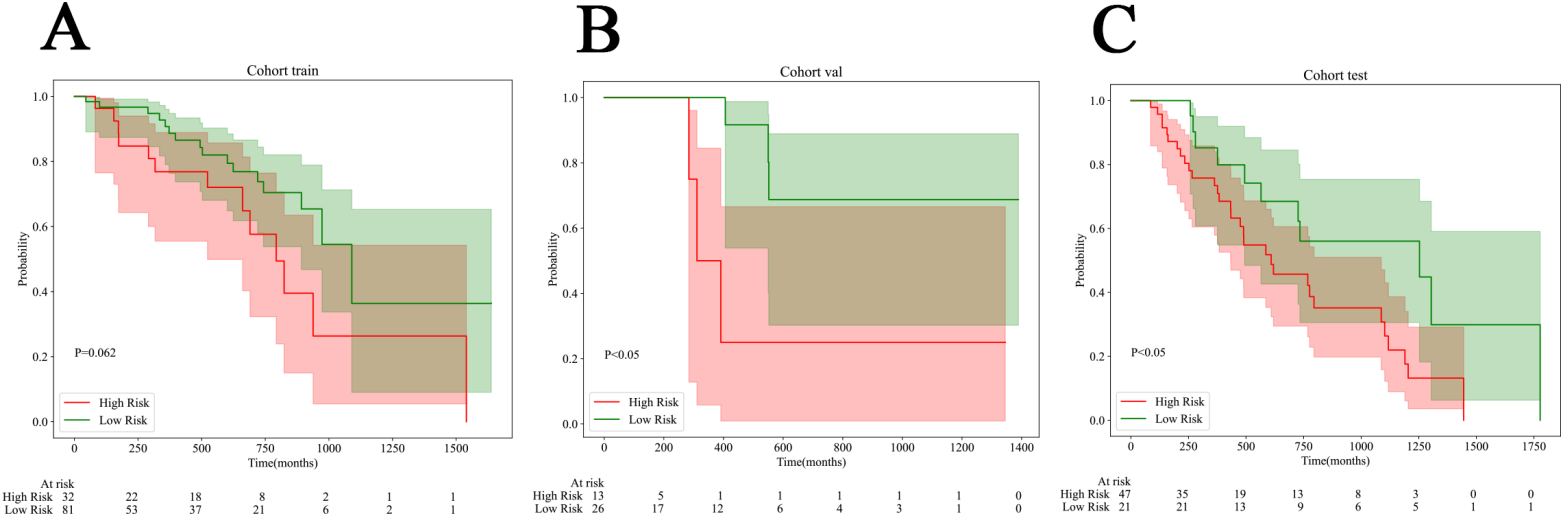
Kaplan-Meier (KM) curves for all cohorts. **(A)** The train cohort. **(B)** The internal validation cohort. **(C)** The external test cohort.

Survival data were collected from a subset of samples from the radiomics & pathomics cohorts, specifically the train set (n=113), the internal validation set (n=39), and the external test set (n=68). The calculation of overall survival (OS) began with the CT examination time recorded in prior research. Every patient underwent telephone follow-up, and the timing of their demise was confirmed through these follow-ups. The optimal cut-off value for survival samples was determined using Nomogram based on xtile. Subsequently, we categorized them into high-risk and low-risk groups, and employed the Kaplan-Meier curve to assess the model. We found that in the validation and test set, the log rank test p value was less than 0.05, which proved the effectiveness of Nomogram in predicting risk stratification (**Figure 7**).

## Discussion

Accurately predicting EGFR mutation status is crucial for doctors to select suitable patients for EGFR-TKIs therapy. Moreover, patients with EGFR mutations generally exhibit a lower response rate to immune checkpoint inhibitors (ICIs), particularly those with exon 19 deletions (8). Therefore, determining the EGFR mutation status is also indispensable for screening individuals suitable for immunotherapy. Given the heterogeneity of tumors, the limitations of biopsies, and the high costs associated with gene testing, there is an urgent need for a more effective, simple, inexpensive, and reproducible method to detect EGFR mutation status in NSCLC. In recent years, the effectiveness of multimodal prediction model in risk stratification and treatment response for ovarian cancer, breast cancer and rectal cancer has been confirmed (23–25). In this study, we utilized CT data and pathological slides from patients with NSCLC that were used in routine diagnosis and treatment. We employed 3D deep learning algorithm to extract features, developed and validated the radiomics model, utilized weak supervised deep learning and multi-instance learning to refine the pathomics model, conducted an in-depth analysis of clinical features using several machine learning techniques, resulting in the formulation of clinical signature and ultimately achieved innovatively Nomogram through the use of fusion algorithms to predict EGFR mutation status and risk stratification in NSCLC. The results indicated that Nomogram exhibited the highest prediction efficiency when compared to the single-mode model. Nomogram enhances the predictive performance of existing models by integrating data from diverse sources and extracting information across various dimensions. This approach had been validated in an external test cohort, demonstrating the robustness and generalizability of Nomogram. The prediction of EGFR mutation status and risk stratification in patients with NSCLC can be realized through multi-disciplinary and multi-dimensional, which addresses the limitations of biopsy tissue acquisition and saves the cost of gene detection. This offers a new perspective and valuable insights for the individualized treatment of patients with NSCLC.

Previous studies on artificial intelligence primarily concentrated on extracting features from a single medical image, potentially underestimating certain characteristics of tumor biology. The features extracted through radiomics originate from a macro perspective, encompassing the spatial distribution and morphological features of the lesion but lacking information at the molecular level. Conversely, pathomics can extract features of microstructures that represent various tissue phenotypes, which can be utilized to predict outcome events. However, it relies on tissue samples, posing the issue of incomplete sampling. By integrating the strengths of both methods, more comprehensive and precise information about tumor tissue can be obtained. In a study predicting the response to immunotherapy in NSCLC, the AUC of the pathomics model reached 0.73, whereas the AUC of the multimodal combined model, integrating radiomics, pathomics, and genomics, was improved to 0.80 (26). A recent study has also confirmed that the combined use of radiomics and pathomics is significantly superior to single models in predicting both the recurrence of early-stage NSCLC and the immunotherapy response in advanced diseases (27). Our research has confirmed the potential benefits of integrating radiomics with pathomics in predicting biomarkers and risk stratification for NSCLC, comprehensively capturing both macro and micro characteristics of tumors, thereby complementing the heterogeneity of tumor tissues. This innovative finding allows for a more comprehensive and accurate prediction of the genetic mutation status of NSCLC patients prior to treatment, thereby guiding the formulation of personalized treatment decisions in clinical practice. Furthermore, it facilitates more targeted management measures for different patients through risk stratification. It also underscored the significance of interdisciplinary integration and the sharing of information among medical institutions. We have made all the codes utilized in this study available for sharing. All workflow and research methods can also be applied to the other diseases besides lung cancer.

Studies that used CT semantic features combined with traditional quantitative radiology features to predict EGFR mutation status were confined to low-order visual features or simplistic high-order features. Deep learning can extract abstract features through multi-layer neural network structure. Several scholars have used 2D CT images as input for neural networks and employed various of models to identify EGFR mutations (18, 19). Despite the promising prediction performance of this method, there was a possibility of losing information and features of lung lesions in three-dimensional position space. The advantage of our study lies in employing a 3D ROI deep learning model, while maintaining the spatial location of the lesion and the accuracy of the extracted information. If the patient has multiple tumor lesions at the same time, our model can assist in the clinical selection of highly suspicious mutation lesions for biopsy. If the gene test result of biopsy tissue is EGFR wild type but the prediction result of this model is EGFR mutation, clinicians may be alerted to the possibility of false negative results due to tumor heterogeneity in the biopsy, necessitating an evaluation of the need for a re-biopsy. As a predictive tool, it offers convenience and reusability without incurring any additional costs.

Previous studies on lung cancer and EGFR mutation primarily concentrated on lung adenocarcinoma (LUAD), as the incidence of EGFR mutation in LUAD was notably the highest (28). However, in actual clinical practice, we encountered a certain percentage of EGFR mutations in lung squamous cell carcinoma (LUSC). The national comprehensive cancer network (NCCN) guideline v1.2021 explicitly states that while the frequency of EGFR mutations in LUSC is lower than that in adenocarcinoma, the expert panel recommends that molecular testing should be considered for all patients with LUSC (7). Hence our study population encompassed multiple histological subtypes of NSCLC, aiming to develop a predictive tool that can be applied to all types of NSCLC, not just LUAD. According to recent studies, patients with NSCLC and EGFR mutations who underwent complete surgical resection and subsequently received oxitinib as targeted therapy had a significantly longer disease-free survival compared to those who were treated with a placebo (29). Simultaneously, the NCCN guidelines recommend that the surgical or biopsy tissues of patients with NSCLC who have undergone complete resection in Stage I B-III A should be considered for molecular detection to determine the need for the use of oxitinib (7). Therefore, this study encompassed not only patients with NSCLC who were in the middle or late stages but also patients who underwent early surgical resection. This offers a foundation for subsequent and preventive treatments for early postoperative patients.

The EGFR mutation status data for all patients in this study were acquired through NGS, which boasts high specificity and sensitivity, rather than immunohistochemical staining, which, despite its high specificity, lacks sensitivity. As a high-throughput sequencing technology, NGS offers the most precise detection of EGFR mutation sites. The most frequent alterations in EGFR are 19del and L858R, with other types of mutations making up only about 10%. There exist some disparities in the choice of targeted therapy and prognosis between EGFR 19del and L858R patients (30). By ANOVA statistical analysis, it indicated that Nomogram was highly accurate in distinguishing all various of EGFR mutation status. This facilitates the selection of therapeutic options for patients with various EGFR mutation subtypes. Through KM curve analysis, it had been demonstrated that Nomogram, which integrated radiomics, pathomics, and clinical features, not only possessed the capability to predict the EGFR mutation status of NSCLC but also exhibited considerable potential in predicting risk stratification. This indicated that CT images and WSIs harbored potential information pertinent to the prognosis of patients. The application of Nomogram facilitates effective pre-treatment prediction of patients’ survival risks, enabling the adoption of more aggressive treatment strategies for potential high-risk patients, while allowing for the judicious avoidance of excessive treatment for low-risk patients.

In recent years, the rapid development of digital pathology using computers has played a crucial role in modern clinical practice. Due to its enhanced network compatibility, ease of storage, and sharability, WSI has surpassed the constraints of traditional microscope slides and human cognition, significantly advancing the clinical application of digital pathology and fostering continuous advancements in knowledge utilization and integration (31). The WSI of an H&E-stained pathological tissue section encompasses a multitude of information regarding tumor cell morphological features, the microenvironment, and immune properties. By leveraging deep learning techniques to identify complex visual features in digital high-resolution histopathological images, significant promise has been demonstrated in predicting various clinical outcomes, such as staging, lymph node metastasis, and treatment response (23, 26, 32). In the past, pathomics research necessitated complex manual annotation at the pixel level by pathologists, which entailed classifying the minute tiles within each WSI (33, 34). Coudray et al. obtained WSIs from the cancer genome atlas (TCGA) for deep CNN training (inception V3), which confirmed that six frequently mutated genes in LUAD are predictable with AUCs ranging from 0.733 to 0.856 (34). Inception_v3 was among the models we selected for patch-level verification and evaluation. Our findings revealed that the DenseNet121 model in patch level and the SVM model in WSI level exhibited significant effectiveness for differentiating tumor and non-tumor regions. Several researchers have discovered that employing weakly supervised deep learning on WSI can yield astonishing outcomes that are comparable to those achieved by supervised deep learning (35). In recent years, the model classification performance based on MIL in the histopathological WSI of many organs has demonstrated remarkable results (36–39). We used WSIs as the sole input without requiring the involvement of pathologists, which avoided extensive pixel-level manual annotation, ultimately leading to significant time and financial savings. Moreover, our model’s performance has been validated across various medical institutions, despite differences in staining protocols and the digital scanners used to obtain WSI. The AUC of pathomics model was 0.737 (the internal validation cohort) and 0.753 (the external test cohort). This suggests that EGFR mutations can influence the microscopic morphology and texture of tumor cells, which are imperceptible to the naked eye. Pathomics also serves as an excellent tool, enabling doctors to preliminarily screen individuals who may be candidates for targeted therapy. For those predicted to have a low likelihood of EGFR mutation, costly molecular testing can be spared.

Our research has some limitations. Firstly, this is a retrospective study, which may potentially introduce a degree of bias in the selection of patients. Extensive and larger-scale prospective validation are still required in the future. Secondly, apart from lung adenocarcinoma, the availability of samples for other tissue types, particularly large cell carcinoma and sarcomatoid carcinoma, is rather limited. The reason may be that the mutation rate of EGFR in other tissue types is lower than that in LUAD, and clinicians rarely recommend gene testing, thus necessitating the collection of a larger sample size.

In conclusion, Nomogram which integrates DLRadiomics, Pathomics and clinical features, can serve as a non-invasive biomarker for predicting EGFR mutation status and risk stratification in NSCLC patients and guiding clinical treatment decisions.

## Data Availability

The raw data supporting the conclusions of this article will be made available by the authors, without undue reservation.

## Supplementary material

Supplementary Table 1 Fusion of min, max, mean results. AUC, area under the receiver operating characteristic curve; CI, confidence intervals; PPV, positive predictive value; NPV, negative predictive value.

Supplementary Table 2 ANOVA Statics of prediction results about various EGFR mutation subtypes. Non-EGFR= EGFR wild-type, EGFR-19 = 19Del (the deletion of exon 19), EGFR-21=L858R (the point mutation of exon 21).
