# Supplementary material for: Enhanced prediction of gene mutation and risk stratification in non-small-cell lung cancer through dual-pathway fusion of radiomics and pathomics
Source: Front Oncol. 2025 Sep 3;15:1646851. doi: 10.3389/fonc.2025.1646851 (PMC12440985; doi:10.3389/fonc.2025.1646851)
Supplement: Supplementary Table 1 — Fusion of min, max, mean results. AUC, area under the receiver operating characteristic curve; CI, confidence intervals; PPV, positive predictive value; NPV, negative predictive value. [file SupplementaryFile1.docx]

## Supplementary Material

### 1A. Radiomics Training Details

**Hyperparameters**: Due to the lack of publicly available pretrained parameters for 3D models, our models were initialized randomly. Considering the limited availability of image data, we employed a cosine annealing learning rate schedule, defined as:

$$\eta_{t}=\eta_{min}^{i}+\frac{1}{2}\left( \eta_{max}^{i}-\eta_{min}^{i} \right)\left( 1+cos\left( \frac{T_{cur}}{T_{i}}\pi\right) \right)$$

The parameters were set as follows: $\eta_{min}^{i}=0$, $\eta_{max}^{i}=0.001$, and $T_{i}=48$, representing the minimum learning rate, the maximum learning rate, and the number of iteration epochs, respectively. The batch size was 4. Other key hyperparameters include the use of SGD (Stochastic Gradient Descent) as the optimizer, and softmax cross-entropy as the loss function, to optimize the training process and ensure effective learning.

### 1B. Details of Fusion Model

we compared the results of the three models using three fusion methods: Mean, Min, and Max, as shown in Table S1. Figure S1 presents the ROC of fusion results.

Table S1. Fusion of min, max, mean results.

| Method | Acc | AUC | 95% CI | Sensitivity | Specificity | PPV | NPV | Youden | Cohort |
| --- | --- | --- | --- | --- | --- | --- | --- | --- | --- |
| mean | 0.921 | 0.965 | 0.9352-0.9939 | 0.930 | 0.915 | 0.892 | 0.945 | 0.412 | train |
| max | 0.897 | 0.934 | 0.8934-0.9743 | 0.915 | 0.883 | 0.855 | 0.933 | 0.577 | train |
| min | 0.915 | 0.965 | 0.9366-0.9928 | 0.901 | 0.926 | 0.901 | 0.926 | 0.203 | train |
| mean | 0.738 | 0.745 | 0.6211-0.8693 | 0.714 | 0.757 | 0.690 | 0.778 | 0.497 | val |
| max | 0.723 | 0.758 | 0.6357-0.8798 | 0.643 | 0.784 | 0.692 | 0.744 | 0.808 | val |
| min | 0.662 | 0.698 | 0.5687-0.8270 | 0.893 | 0.486 | 0.568 | 0.857 | 0.021 | val |
| mean | 0.568 | 0.677 | 0.5572-0.7973 | 0.846 | 0.464 | 0.373 | 0.889 | 0.308 | test |
| max | 0.642 | 0.642 | 0.5143-0.7700 | 0.654 | 0.638 | 0.405 | 0.830 | 0.488 | test |
| min | 0.621 | 0.654 | 0.5230-0.7859 | 0.769 | 0.565 | 0.400 | 0.867 | 0.265 | test |

Note. – AUC= area under the receiver operating characteristic curve, CI= confidence intervals, PPV= positive predictive value, NPV= negative predictive value.


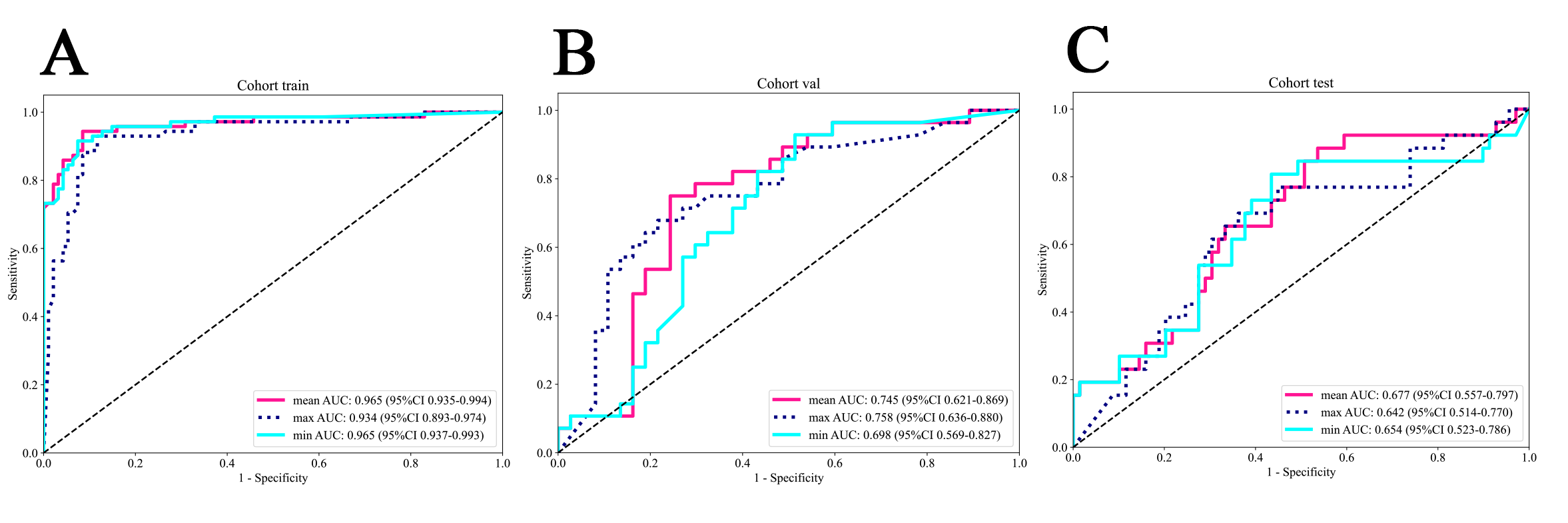


Fig S1. The ROC of fusion results. (a) The train cohort. (b) The internal validation cohort. (c) The external test cohort.

### 2A. Pathomics Training Details

**Hyperparameters**: To enhance the generalization capabilities of our pathology model, we meticulously adjusted the learning rate using a cosine decay algorithm. This approach is characterized as follows:

$$\eta_{t}=\eta_{min}^{i}+\frac{1}{2}\left( \eta_{max}^{i}-\eta_{min}^{i} \right)\left( 1+cos\left( \frac{T_{cur}}{T_{i}}\pi\right) \right)$$

In this formulation, $\eta_{min}^{i}=0$ sets the minimum learning rate, $\eta_{max}^{i}=0.01$ establishes the maximum learning rate, and $T_{i}=50$ denotes the number of epochs in the iterative training process. This learning rate schedule allows for gradual reduction of the learning rate, facilitating fine-tuning of the model as training progresses.

To optimize the training process and enhance predictive accuracy, we employed Stochastic Gradient Descent (SGD) as the optimizer, which is known for its efficiency and effectiveness in handling large datasets. Additionally, we used softmax cross-entropy as the loss function to compute the probability distribution over the target classes, enabling the model to make accurate predictions.

### 2B. Multi-Instance Learning-Based Feature Fusion

In this study, we employed a multi-instance learning-based approach for feature fusion, aiming to enhance the predictive accuracy of our models. This method involves integrating various data points or instances from a single sample to formulate a comprehensive feature set. Such an approach is crucial for the effective analysis and prediction of complex clinical outcomes. Below, we outline the specific steps and techniques utilized in this feature fusion process:

1. **Patch Prediction**: We utilized the resnet50 model to predict each patch, obtaining corresponding probabilities and labels, denoted as $Patch_{prob}$ and $Patch_{pred}$, respectively. The prediction probabilities were retained to two decimal places.
2. **Multi Instance Learning Feature Aggregation:**
   1. **Histogram Feature Aggregation**:
      1. We treated each distinct number as a "bin" and counted the occurrence of each type of data across these bins.
      2. The frequencies of $Patch_{prob}$ and $Patch_{pred}$ falling into each bin were tallied.
      3. All features underwent min-max normalization.
      4. This process resulted in the generation of $Histo_{prob}$ and $Histo_{pred}$.
   2. **Bag of Words (BoW) Feature Aggregation**:
      1. Initially, a dictionary was created by identifying unique elements within $Patch_{prob}$ and $Patch_{pred}$.
      2. Each patch was then represented as a vector, where the frequency of each dictionary element in the patch was noted.
      3. We applied Term Frequency-Inverse Document Frequency (TF-IDF) transformation to these vectors, emphasizing the importance of less frequent but more informative features.
      4. This resulted in a BoW feature representation for each patch, encapsulating both the presence and significance of features within a patch.
      5. The final BoW features, denoted as $BoW_{prob}$ and $BoW_{pred}$, offered a comprehensive and weighted representation of the patches, suitable for subsequent analytical processes.
3. **Feature Early Fusion**: The final stage in our multi-instance learning-based feature fusion involves the integration of the previously derived features: $Histo_{prob}$, $Histo_{pred}$, $Bow_{prob}$, and $Bow_{pred}$. To achieve this, we employ a feature concatenation method, symbolized by $\oplus$, which combines these individual feature sets into a single, comprehensive feature vector. The specific formula for this concatenation is as follows:

$$feature_{fusion}=Histo_{prob}\oplus Histo_{pred}\oplus Bow_{prob}\oplus Bow_{pred}$$

### 3A. Distribution of predictive features for Nomogram across different EGFR mutation subtypes.

Through ANOVA statistical analysis, as shown Table S2, we found that the combined Nomogram model has a p-value less than 0.05 in different groups, demonstrating that our model can effectively predict various EGFR mutation status. Figure S2 presents prediction distribution of different signatures in all cohorts about various EGFR mutation subtypes.

Table S2. ANOVA Statics of prediction results about various EGFR mutation subtypes.

| Signature | ALL | EGFR-19 | EGFR-21 | EGFR-Others | Non-EGFR | pvalue | group |
| --- | --- | --- | --- | --- | --- | --- | --- |
| Clinical | 0.512±0.169 | 0.584±0.096 | 0.637±0.111 | 0.625±0.153 | 0.415±0.156 | <0.001 | train |
| DLRadiomics | 0.493±0.450 | 0.885±0.247 | 0.895±0.231 | 0.806±0.253 | 0.126±0.259 | <0.001 | train |
| Pathomics | 0.498±0.402 | 0.836±0.193 | 0.898±0.073 | 0.906±0.058 | 0.135±0.172 | <0.001 | train |
| Nomogram | 0.491±0.387 | 0.823±0.151 | 0.873±0.143 | 0.847±0.116 | 0.146±0.177 | <0.001 | train |
| Clinical | 0.548±0.161 | 0.548±0.158 | 0.651±0.132 |  | 0.494±0.156 | 0.02 | val |
| DLRadiomics | 0.392±0.419 | 0.658±0.463 | 0.452±0.376 |  | 0.268±0.391 | 0.061 | val |
| Pathomics | 0.572±0.322 | 0.826±0.132 | 0.664±0.290 |  | 0.435±0.319 | 0.004 | val |
| Nomogram | 0.465±0.258 | 0.659±0.168 | 0.569±0.248 |  | 0.344±0.228 | 0.002 | val |
| Clinical | 0.430±0.138 | 0.503±0.188 | 0.418±0.164 | 0.376 | 0.425±0.133 | 0.591 | test |
| DLRadiomics | 0.369±0.214 | 0.511±0.216 | 0.536±0.268 | 0.889 | 0.340±0.196 | 0.007 | test |
| Pathomics | 0.126±0.093 | 0.216±0.120 | 0.155±0.100 | 0.072 | 0.117±0.087 | 0.075 | test |
| Nomogram | 0.135±0.040 | 0.187±0.053 | 0.175±0.050 | 0.253 | 0.127±0.031 | <0.001 | test |

Note. – Non-EGFR= EGFR wild-type, EGFR-19=19Del (the deletion of exon 19), EGFR-21=L858R (the point mutation of exon 21).


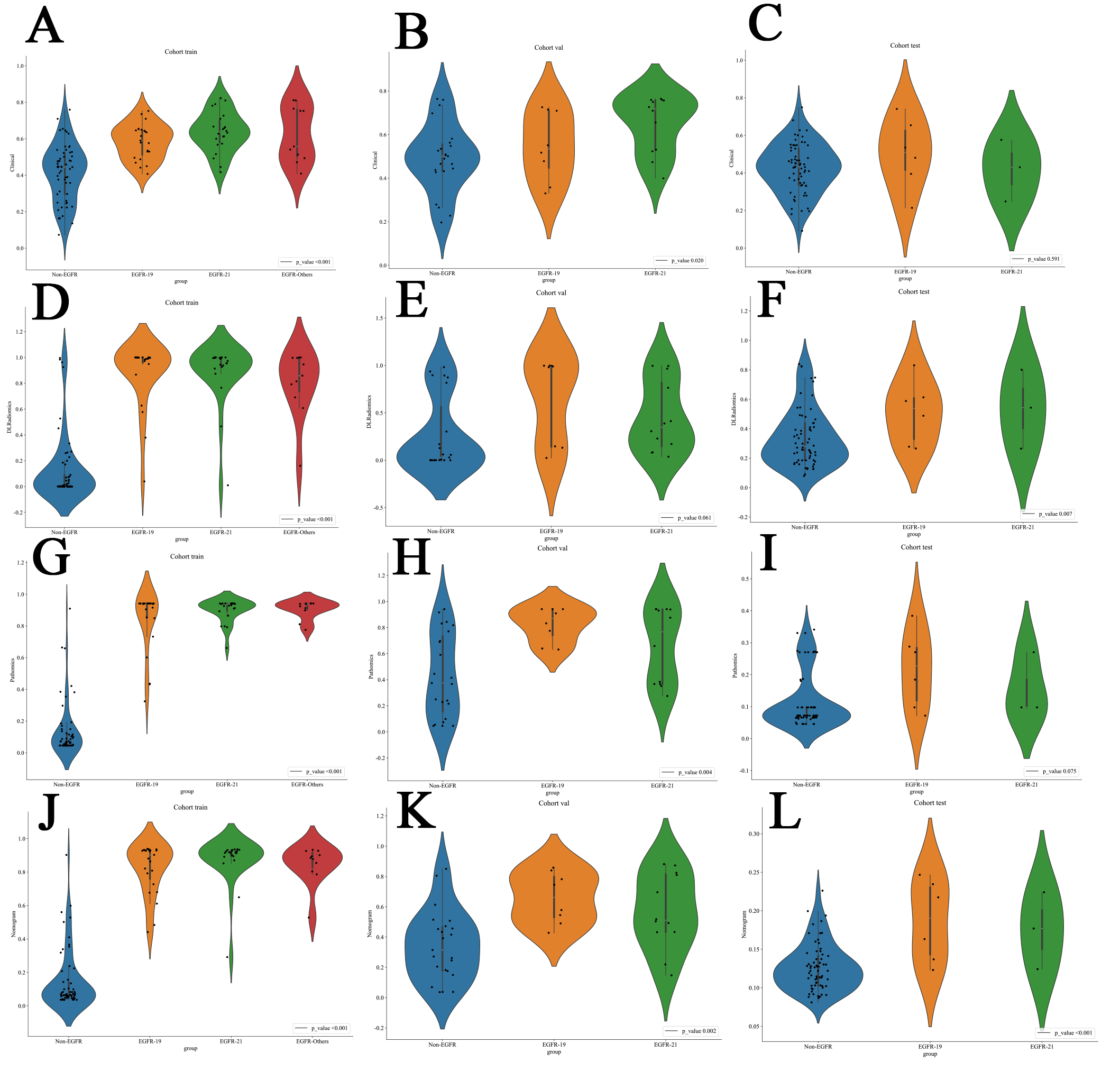


Fig S2. Prediction Distribution of different signature in all cohorts about various EGFR mutation subtypes. (a) Clinical in the train cohort. (b) Clinical in the internal validation cohort. (c) Clinical in the external test cohort. (d) DLRadiomics in the train cohort. (e) DLRadiomics in the internal validation cohort. (f) DLRadiomics in the external test cohort. (g) Pathomics in the train cohort. (h) Pathomics in the internal validation cohort. (i) Pathomics in the external test cohort. (j) Nomogram in the train cohort. (k) Nomogram in the internal validation cohort. (l) Nomogram in the external test cohort. Non-EGFR, EGFR wild-type; EGFR-19, 19Del (the deletion of exon 19); EGFR-21, L858R (the point mutation of exon 21).

### 3B. Combined Model.

We integrated all clinical features with the signature to construct a Combined Model, which facilitates clinical utilization. Figure S3 shows the combined model for clinical use in predicting EGFR status for the patients with NSCLC.


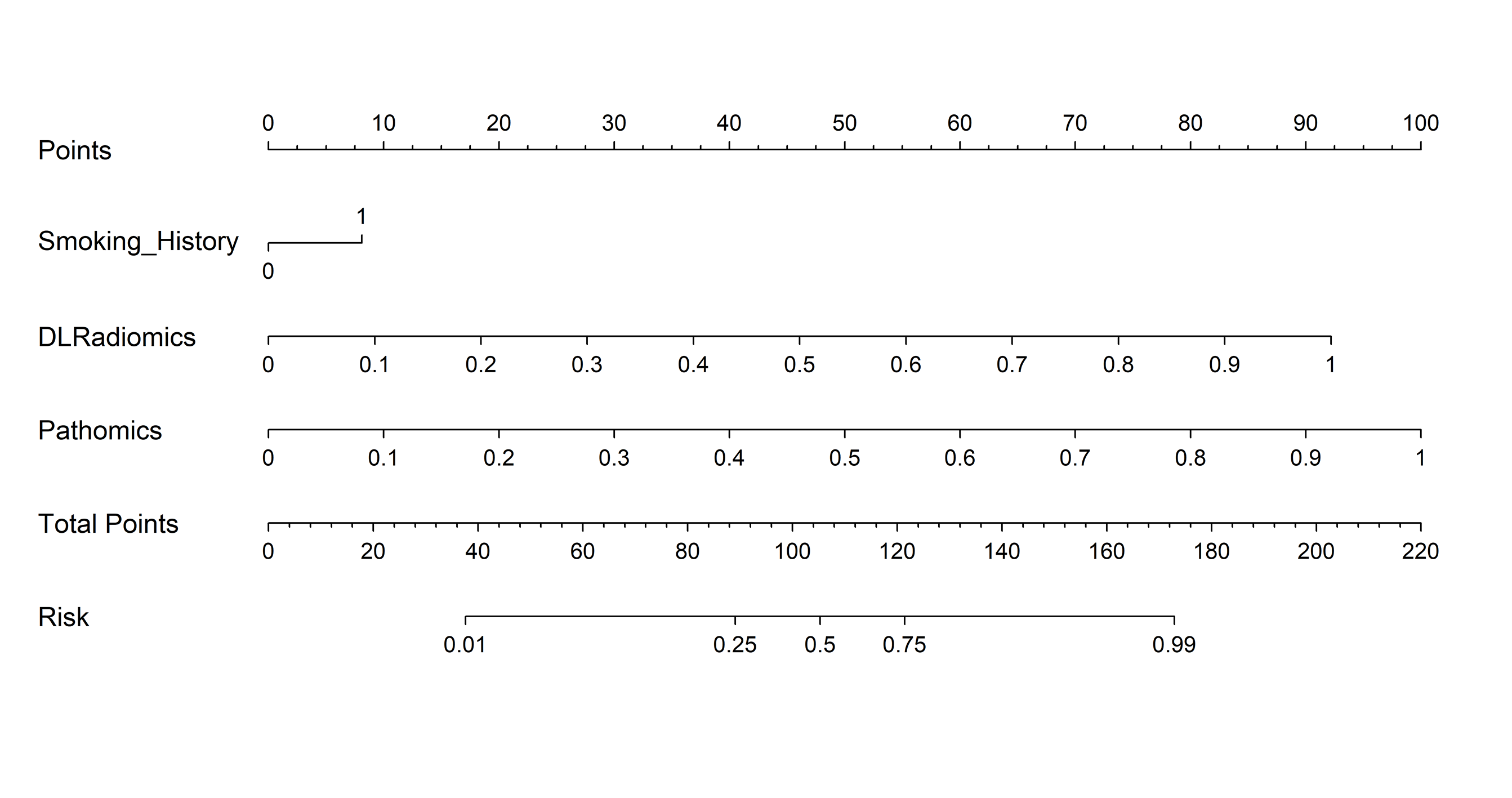


Fig S3. The Combined Model for clinical use in prediction of EGFR status.
